# Dietetic Treatment of Dyspepsia

**Published:** 1885-03

**Authors:** 


### THE DIETETIC TREATMENT OF DYSPEPSIA.

Dr. Austin Flint, Sr., of New York, recently read a paper on this subject.

The first point which he wished to make was, he said, that dyspepsia denoted an affection distinct from indigestion. Having described certain of the symptoms of dyspepsia, he remarked that it was, in many cases, referable to the mind, a fact which he believed he had been the first to call attention to, in a paper published in the *American Journal of the Medical Science* in 1842. He then spoke of the fallacies which had formerly been entertained, more particularly in New England, in regard to the advantages of a severe regimen and restricted diet. At the present day, dyspepsia was much less frequent than in past years, and those who did suffer from it were mostly those who endeavored to regulate their diet and habits in accordance with the notions that formerly prevailed. One very prolific source of trouble was the practice of introspection thus engendered.

In the treatment of dyspepsia the patient was to be encouraged to forget his strict habits of eating. No rigid system was to be observed, and food in sufficient quantity and variety was essential· The guides for eating should be the appetite, the palate, and common sense. The cravings of nature were not to be disregarded. The patient's experience with certain articles of food was apt to be extremely fallacious, and he should not be guided by this, for what at one time might disagree with him, at another might give rise to no trouble whatever. Dietetic idiosyncrasies were vastly less numerous than was generally supposed to be the case. The instructions to such a patient, therefore, should be something like the following: Do not adopt the rule of eating only at stated intervals, but eat whenever you desire food. Be doubtful of your past experience, and eat whatever is most acceptable to your palate. Try to have good cooking and don't leave the table until your hunger is perfectly satisfied. Eat animal as well as vegetable food, and take as much fluid as thirst dictates. In general, the diet which was beneficial to the healthy individual was beneficial to the dyspeptic. It was a fallacy to suppose that the organs of digestion required periods of prolonged rest. If allowed to rest, their functions would become perverted. Is was of great importance that the dyspeptic should occupy his mind with other classes of objects than his food. Practical lessons on this subject might be learned from the gourmand, who, whatever else he might suffer, seldom had dyspepsia; and the same was true, on the other hand, of the hard-working mechanic, who, instead of living to eat, eats to live.—*Medical News.*

